# Initial Clip Selection and Outcomes After Mitral Transcatheter Edge-to-Edge Repair: A Single-Center Experience

**DOI:** 10.3390/jcm15208176

**Published:** 2026-10-21

**Authors:** Aysel Akhundova, Berhan Keskin, Umeyir Savur, Özlem Önder, Ebubekir Yiğitbay, Ekrem Guler, Haci Murat Güneş, Mehmet Emir Arman, Bilal Boztosun

**Affiliations:** 1 Department of Cardiology, Medipol Mega University Hospital, Istanbul Medipol University, 34214 Istanbul, Turkey; 2 Internal Medicine, Ascension St Vincent Hospital—Indianapolis, Indianapolis, IN 46260, USA

**Keywords:** transcatheter edge-to-edge repair, mitral regurgitation, MitraClip, clip selection, residual mitral regurgitation, transmitral gradient, mitral valve

## Abstract

**Background:** Whether the initial clip type selected during transcatheter edge-to-edge repair (TEER) of the mitral valve—grasping width and arm length—independently influences procedural and echocardiographic outcomes remains uncertain. **Methods:** We retrospectively analyzed 138 consecutive patients undergoing mitral TEER. The first implanted clip was classified as narrow standard-length (NT), long narrow-arm (XT), or long wide-arm (XTW). Determinants of initial clip selection and its association with procedural success (residual mitral regurgitation [MR] ≤ 1+), post-procedural transmitral gradient, and the need for ≥2 clips were assessed using multivariable logistic and linear regression. **Results:** Median age was 74.0 (66.0–80.0), 74.5 (66.0–82.5), and 75.0 (70.0–79.0) years, and median LVEF was 35.0% (30.0–50.0), 30.0% (25.0–45.0), and 40.0% (25.0–45.0), for the NT, XT, and XTW groups, respectively; 66% of patients overall were male. An XTW clip was implanted first in 61.6% of patients. XTW selection was independently associated with larger effective regurgitant orifice area, lower LVEF, longer posterior leaflet length, and secondary MR (all *p* < 0.05), whereas NT selection favored younger age, smaller orifice, and higher LVEF. After adjustment for these factors, no statistically significant independent association was detected between initial clip type and residual MR ≥2+, post-procedural transmitral gradient, or the need for additional clips (all *p* > 0.05); however, confidence intervals were wide. On univariable analysis, larger left atrial size and moderate-to-severe tricuspid regurgitation were associated with residual MR ≥ 2+, but neither remained significant after multivariable adjustment. **Conclusions:** Initial clip selection was associated with valve anatomy, LVEF and MR etiology. No statistically significant independent association between initial clip type and procedural or short-term echocardiographic outcomes was detected in this cohort. Given the observational design, confounding by indication, limited sample size and wide confidence intervals, clinically relevant effects cannot be excluded, and these findings do not establish the superiority or equivalence of any clip-selection strategy.

## 1. Introduction

Mitral regurgitation (MR) is the most common valve disease, and severe MR is associated with substantial morbidity, heart failure hospitalization, reduced functional capacity, and increased mortality when left untreated. Both primary (degenerative) MR, resulting from structural leaflet or chordal abnormalities, and secondary (functional) MR, related to left ventricular remodeling and annular dilatation or atrial dilatation, represent important clinical entities with distinct pathophysiological mechanisms and therapeutic considerations [1]. Surgical mitral valve repair or replacement remains the standard treatment for selected patients; however, a considerable proportion of patients are deemed inoperable or at high surgical risk because of advanced age, frailty, left ventricular dysfunction, or multiple comorbidities [2].

Over the last decade, transcatheter edge-to-edge repair (TEER) has emerged as an established minimally invasive treatment option for symptomatic patients with severe MR who are not suitable candidates for conventional surgery. Since the introduction of MitraClip, multiple studies and registries have demonstrated the safety and efficacy of TEER in reducing MR severity, improving symptoms, and decreasing heart failure events in appropriately selected patients [3]. Continuous technological refinement has further expanded the applicability of TEER, allowing treatment of increasingly complex mitral anatomies and a broader spectrum of patient profiles.

The availability of contemporary clip systems with different arm lengths and widths has introduced new procedural strategies tailored to leaflet anatomy, coaptation gap, jet location, flail width, and valve area [4]. In current practice, operators may choose among short, long, narrow, or wide clip configurations to optimize leaflet grasping and maximize MR reduction while minimizing transmitral gradients. Although this device’s versatility represents a major advance, evidence guiding optimal initial clip selection in routine clinical practice remains limited. Procedural decision-making is frequently based on operator experience and anatomical judgment rather than robust comparative data.

Recent registry data have suggested that clip selection may influence procedural efficiency and residual MR, particularly in specific etiologic subsets such as primary versus secondary MR. However, real-world data evaluating the association between initial clip type and both procedural and mid-term clinical outcomes remain scarce, especially from high-volume centers outside large multicenter registries [2].

This study aimed to identify the anatomical and echocardiographic factors associated with initial clip selection and to evaluate the associations between initial clip type and procedural outcomes, including residual mitral regurgitation and post-procedural transmitral mean gradient.

## 2. Methods

### 2.1. Study Design and Population

This was a retrospective, observational, single-center cohort study of 138 consecutive patients who underwent transcatheter edge-to-edge repair (TEER) for significant mitral regurgitation (MR) at our institution between January 2021 and January 2024. A dedicated multidisciplinary Heart Team of interventional cardiologists, cardiac surgeons, imaging specialists, heart failure specialists, and anesthesiologists evaluated all procedures.

Patients were considered eligible for TEER if they had symptomatic moderate-to-severe or severe MR despite guideline-directed medical therapy and were judged to be at high or prohibitive surgical risk, or were otherwise deemed suitable for transcatheter treatment on the basis of clinical status and anatomical feasibility. Both primary (degenerative) and secondary (functional) MR etiologies were included; MR etiology was adjudicated according to contemporary echocardiographic criteria and Heart Team consensus. Patients with prior mitral valve surgery, active infective endocarditis, intracardiac thrombus, severe mitral stenosis, or insufficient imaging quality precluding procedural guidance were excluded. The study was conducted in accordance with the Declaration of Helsinki and was approved by the local institutional ethics committee. This study is reported in accordance with the STROBE (Strengthening the Reporting of Observational Studies in Epidemiology) guideline for cohort studies.

### 2.2. Echocardiographic Assessment

All patients underwent comprehensive transthoracic and transesophageal echocardiography before the procedure. Baseline assessment included left ventricular ejection fraction (LVEF), left ventricular dimensions, left atrial size, pulmonary artery systolic pressure, tricuspid regurgitation severity, mitral valve area, leaflet anatomy, coaptation gap, flail width/gap when present, and MR severity. MR severity was graded using an integrative multiparametric approach incorporating vena contracta width, effective regurgitant orifice area (EROA), regurgitant volume, pulmonary vein flow pattern, and qualitative Doppler parameters, in accordance with current guideline recommendations [2]. Complex mitral anatomy was defined a priori on pre-procedural transesophageal echocardiography [5] as the presence of at least one of the following: large flail gap (≥10 mm) and/or flail width (≥15 mm); commissural lesion or commissural jet origin; calcification in the grasping zone; leaflet cleft or deep indentation in the target segment; short posterior leaflet (length < 10 mm); multiple or broad regurgitant jets involving more than one segment; small baseline mitral valve area (<4.0 cm^2^) and/or baseline mean transmitral gradient ≥ 4 mmHg; and Barlow disease or extensive bileaflet prolapse.

Transthoracic echocardiography was repeated before discharge and during outpatient follow-up at the treating physician’s discretion. Post-procedural residual MR and transmitral mean gradient were assessed by transthoracic echocardiography before hospital discharge, and this examination was used to define all procedural echocardiographic outcomes in the present analyses. Follow-up echocardiograms, performed at the discretion of the treating physicians, were not used for the endpoint analyses. Residual MR was graded using an integrative multiparametric approach according to ASE recommendations for the evaluation of valvular regurgitation after percutaneous valve repair [6]. Transmitral mean gradient was measured by continuous-wave Doppler and averaged over 3 cardiac cycles in sinus rhythm and 5 cycles in atrial fibrillation.

### 2.3. Procedure and Clip Selection

All TEER procedures were performed in a catheterization laboratory or structural heart suite under general anesthesia or deep sedation with fluoroscopic and real-time transesophageal echocardiographic guidance, using the MitraClip G4 system (Abbott Vascular, Santa Clara, CA, USA) in all patients. Transseptal puncture was performed using standard technique, followed by advancement of a 24-F guiding catheter into the left atrium. The initial clip type was selected based on pre-procedural and intra-procedural imaging findings, including leaflet length, coaptation depth, jet origin, flail segment dimensions, annular geometry, valve area, and the anticipated risk of post-procedural mitral stenosis. After grasping and closure, clip position and residual MR were assessed by transesophageal echocardiography and transmitral gradient measurement before release; an additional clip was implanted if MR reduction was judged insufficient. Optimal procedural MR reduction was defined as a residual MR grade ≤ 1+.

For analytic purposes, the first implanted clip in each patient was categorized by grasping width and arm length as narrow, standard-length (NT), long, narrow-arm (XT), or long, wide-arm (XTW), consistent with the device nomenclature used throughout the results. The MitraClip G4 system is available in four configurations (NT, NTW, XT and XTW). In the present cohort, NTW was not used as the initial device; NTW clips were used only as additional clips in 2 patients; therefore, the first implanted clip was classified as NT, XT or XTW. Clinically, additional clips could be implanted to further optimize MR reduction.

### 2.4. Study Endpoints

The primary procedural endpoint was procedural success, defined according to Mitral Valve Academic Research Consortium criteria as successful device implantation with a significant reduction in MR (residual MR ≤ 1+) and no procedural mortality or need for emergency surgery. Because no procedural deaths or emergency surgical interventions occurred in the cohort, the predefined composite procedural-success endpoint was determined entirely by the achievement of residual MR ≤ 1+. Accordingly, residual MR ≥ 2+ represented failure to achieve the predefined procedural-success endpoint and was used as the dependent variable in the regression analyses. Procedural echocardiographic outcomes, including residual MR and transmitral mean gradient, were assessed by pre-discharge transthoracic echocardiography. Follow-up echocardiographic measurements were not included in the outcome analyses.

Secondary endpoints included heart failure rehospitalization, mitral valve reintervention, stroke, major bleeding, and peripheral vascular complications.

In-hospital complications were defined according to the Mitral Valve Academic Research Consortium (MVARC) criteria [7]. Acute kidney injury was defined as an increase in serum creatinine of ≥0.3 mg/dL (≥26.5 μmol/L) or ≥50% from baseline within 7 days after the procedure, in accordance with the MVARC (modified KDIGO/AKIN) criteria [8]. Major bleeding was defined according to the MVARC bleeding definitions as overt bleeding associated with a hemoglobin drop of ≥3 g/dL, transfusion of ≥2 units of red blood cells, hospitalization, or permanent injury, or requiring surgical intervention [7]. Cerebrovascular events included stroke and transient ischemic attack, defined according to MVARC as an acute episode of focal or global neurological deficit caused by vascular brain injury, confirmed by a neurologist and/or neuroimaging. Single-leaflet device attachment was defined as attachment of a clip to only one mitral leaflet, identified by echocardiography during or after the procedure; device detachment (embolization) was defined as complete loss of attachment of the clip from both leaflets.

Clinical follow-up data were obtained from outpatient visits, hospital electronic records, and telephone contact when necessary; follow-up duration was calculated from the index TEER procedure to the last available clinical contact or death. Crude, unadjusted rates of all-cause mortality and cardiovascular death within 2 years were also recorded and are reported descriptively as exploratory, hypothesis-generating observations; a formal, adjusted time-to-event analysis of mortality was not performed and was outside the scope of this study.

### 2.5. Statistical Analysis

Continuous variables were tested for normality using the Shapiro–Wilk test and are presented as mean ± standard deviation or median (interquartile range) according to distribution; categorical variables are presented as frequency (percentage). Comparisons among the three initial-clip-type groups (NT, XT, and XTW) were performed using one-way ANOVA or the Kruskal–Wallis test for continuous variables, and the chi-square or Fisher exact test for categorical variables, as appropriate. Pre-discharge residual MR grade and transmitral mean gradient were available in all patients, and there were no missing data for the variables included in the regression models; all analyses were therefore performed in the full cohort of 138 patients.

Factors associated with initial clip selection were evaluated using two separate binary logistic regression models: XTW versus NT or XT, and NT versus XT or XTW. These comparisons were selected because of the relatively small numbers of patients receiving NT and XT clips, which raised concerns regarding parameter burden and estimate instability in a multinomial model. XT was therefore included in the respective comparison groups rather than modelled as a separate outcome category. Accordingly, these analyses were intended to identify factors associated with XTW and NT selection and did not constitute a formal overall comparison among the three clip types. Candidate predictors, selected on clinical grounds, included age, sex, EROA, LVEF, posterior leaflet length, complex mitral valve anatomy, MR etiology (secondary vs. primary), baseline transmitral mean gradient, and mitral annular calcification; continuous variables were entered as clinically meaningful increments (age per 5 years, EROA per 5 mm^2^ increase, LVEF per 5 percentage points, posterior leaflet length per 1 mm, and transmitral mean gradient per 1 mmHg). Pre-discharge residual MR grade and pre-discharge transmitral mean gradient were available in all patients.

Predictors of residual MR ≥ 2+ after TEER were first screened using univariable logistic regression. Variables that were clinically relevant, together with those significant on univariable analysis, were entered into multivariable models. Left atrial diameter and left atrial area were strongly correlated; to avoid collinearity, only left atrial diameter, which showed the stronger univariable association with residual MR ≥ 2+, was entered into the multivariable models.

NT-proBNP was not considered a candidate predictor of initial clip selection because it does not directly inform the anatomical choice of device and largely reflects left ventricular dysfunction and MR etiology, which were already included in the models. In the outcome analyses, log-transformed NT-proBNP was evaluated in univariable analysis and was not associated with residual MR ≥ 2+. Because the effect of initial clip strategy was evaluated using two alternative binary definitions, two parallel multivariable models were constructed (Model 1: initial XTW vs. other clip types; Model 2: initial NT vs. other clip types), each adjusted for LVEF, MR etiology, complex mitral anatomy, EROA, left atrial diameter, moderate-to-severe tricuspid regurgitation (TR), baseline transmitral mean gradient, posterior leaflet length, and number of implanted clips. Model discrimination was assessed using the area under the receiver-operating-characteristic curve (AUC) and overall predictive accuracy using the Brier score; internal validation was performed with 1000 bootstrap resamples to derive optimism-corrected AUC and Brier scores. Multicollinearity among predictors was assessed using variance inflation factors (VIFs).

Factors associated with post-procedural transmitral mean gradient were evaluated using multivariable linear regression, with the same two clip-type coding strategies (Model 1: initial XTW vs. other; Model 2: initial NT vs. other) and adjustment for LVEF, MR etiology, complex mitral anatomy, EROA, left atrial diameter, moderate-to-severe TR, baseline transmitral mean gradient, posterior leaflet length, and number of implanted clips; regression coefficients (β) with 95% confidence intervals (CIs) are reported. An analogous pair of multivariable logistic regression models, adjusted for LVEF, MR etiology, complex mitral anatomy, EROA, left atrial diameter, moderate-to-severe TR, baseline transmitral mean gradient, and posterior leaflet length, was used to identify predictors of the need for ≥2 implanted clips.

Results are presented as adjusted odds ratios (ORs), regression coefficients (β), or unadjusted ORs with 95% CIs, as appropriate. All tests were two-sided, and a *p*-value < 0.05 was considered statistically significant. Statistical analyses were performed using IBM SPSS Statistics version 26 (IBM Corp., Armonk, NY, USA).

As a sensitivity analysis, residual MR ≥ 2+ and post-procedural transmitral mean gradient were also modelled with initial clip type as a three-level categorical variable (XT as reference), excluding the number of implanted clips; global *p*-values were derived from the joint test of clip-type terms.

## 3. Results

### 3.1. Baseline Characteristics

Among the 138 patients included, the first implanted clip was NT in 25 patients (18.1%), XT in 28 patients (20.3%), and XTW in 85 patients (61.6%). Baseline demographic and clinical characteristics were generally well balanced across the three groups (Table 1). Median age was similar (74.0, 74.5, and 75.0 years for NT, XT, and XTW, respectively; *p* = 0.827), as were the prevalence of hypertension, diabetes, coronary artery disease, atrial fibrillation, and prior heart failure hospitalization (all *p* > 0.05). Among baseline medications, angiotensin receptor–neprilysin inhibitor (ARNI) use (20.0% vs. 53.6% vs. 24.7%; *p* = 0.008) and mineralocorticoid receptor antagonist use (60.0% vs. 71.4% vs. 45.9%; *p* = 0.049) differed significantly across NT, XT, and XTW groups, respectively, and NT-proBNP levels were lower in the NT group (median 2775 pg/mL) than in the XT (13,727 pg/mL) and XTW (8897 pg/mL) groups (*p* = 0.014), which is consistent with a greater heart-failure burden, rather than as evidence supporting it. Because approximately 30 baseline variables were compared without correction for multiple testing, these nominally significant differences, particularly the borderline difference in MRA use (*p* = 0.049), should be interpreted with caution; none would remain significant after Bonferroni correction. With respect to mitral valve anatomy, EROA was largest in the XTW group (median 50 mm^2^) compared with the XT (43 mm^2^) and NT (45 mm^2^) groups (*p* = 0.003), and complex mitral anatomy tended to be less frequent among XTW recipients (12.9% vs. 32.1% vs. 24.0%; *p* = 0.061). Posterior leaflet length was numerically longer in the XTW group but did not differ significantly (*p* = 0.211). MR etiology distribution, baseline transmitral mean gradient, mitral annular calcification, left ventricular and left atrial dimensions, pulmonary artery systolic pressure, and the prevalence of moderate-to-severe TR were similar across groups (all *p* > 0.05).

### 3.2. Procedural and Follow-Up Outcomes by Initial Clip Type

Procedural and post-procedural characteristics are summarized in Table 2. The mean number of implanted clips per patient was similar across groups (1.72 ± 0.74 for NT, 1.89 ± 0.50 for XT, and 1.64 ± 0.67 for XTW; *p* = 0.195). Optimal procedural MR reduction (residual MR grade 0–1) was achieved in 44.0%, 46.4%, and 49.4% of the NT, XT, and XTW groups, respectively, with no significant between-group difference in the distribution of post-procedural MR grade (Table 2). Post-procedural transmitral mean gradient (*p* = 0.211), post-procedural pulmonary artery systolic pressure (*p* = 0.131), and post-procedural TR grade (*p* = 0.380) also did not differ significantly by initial clip type. Single-leaflet device attachment/detachment was numerically more frequent with XTW (7.1%) than with XT (3.6%) or NT (0%), but this difference did not reach statistical significance (*p* = 0.427).

In-hospital complications, including acute kidney injury, need for dialysis, cerebrovascular accident, major bleeding, and peripheral vascular complication, were comparable across groups (all *p* > 0.05), although major bleeding was numerically more frequent with XTW (16.5%) than with XT (7.1%) or NT (4.0%) (*p* = 0.187). No procedural deaths or emergency surgical interventions occurred in any of the three clip groups. Consequently, the procedural-success rates, according to the predefined composite endpoint, were 44.0%, 46.4%, and 49.4% in the NT, XT, and XTW groups, respectively.

### 3.3. Determinants of Initial Clip Selection

On multivariable logistic regression, selection of an XTW clip was independently associated with larger EROA (adjusted OR 3.869 per 5-unit increase, 95% CI 2.215–6.759; *p* < 0.001), lower LVEF (adjusted OR 0.727 per 5% increase, 95% CI 0.565–0.934; *p* = 0.013), longer posterior leaflet length (adjusted OR 1.526 per 1 mm increase, 95% CI 1.116–2.086; *p* = 0.008), absence of complex mitral anatomy (adjusted OR 0.255 for complex anatomy, 95% CI 0.079–0.825; *p* = 0.023), and secondary rather than primary MR (adjusted OR 12.684, 95% CI 2.559–62.868; *p* = 0.002) (Table 3). Age, sex, baseline transmitral mean gradient, and mitral annular calcification were not independently associated with XTW selection.

Conversely, NT clip selection was independently associated with younger age (adjusted OR 0.768 per 5-year increase, 95% CI 0.590–0.999; *p* = 0.049), smaller EROA (adjusted OR 0.341 per 5 mm^2^ increase, 95% CI 0.182–0.640; *p* < 0.001), and higher LVEF (adjusted OR 1.440 per 5% increase, 95% CI 1.063–1.949; *p* = 0.019). Posterior leaflet length (*p* = 0.064) and secondary MR (*p* = 0.060) showed borderline inverse associations with NT selection, while sex, complex mitral anatomy, baseline transmitral mean gradient, and mitral annular calcification were not independently associated with clip choice. Taken together, these findings indicate that initial clip selection was systematically anatomy- and hemodynamics-driven, favoring wider and longer clips in patients with larger regurgitant orifices, more depressed LVEF, longer posterior leaflets, and secondary MR, and narrower clips in patients with smaller orifices, preserved LVEF, and younger age.

### 3.4. Predictors of Residual Mitral Regurgitation ≥ 2+

On univariable logistic regression, larger left atrial diameter (OR 1.31 per 5 mm increase, 95% CI 1.05–1.64; *p* = 0.016), larger left atrial area (OR 1.46 per 5 cm^2^ increase, 95% CI 1.00–2.11; *p* = 0.047), and moderate-to-severe TR (OR 3.20, 95% CI 1.16–8.84; *p* = 0.025) were significantly associated with residual MR ≥ 2+ after TEER (Table 4). No significant univariable associations were found for age, sex, NT-proBNP, MR etiology, complex anatomy, EROA, left ventricular dimensions or LVEF, baseline transmitral mean gradient, posterior leaflet length, mitral annular calcification, number of implanted clips, or initial clip type (initial XTW vs. other: OR 0.85, 95% CI 0.43–1.69, *p* = 0.637; initial NT vs. other: OR 1.21, 95% CI 0.51–2.89, *p* = 0.672).

**Figure 1:**
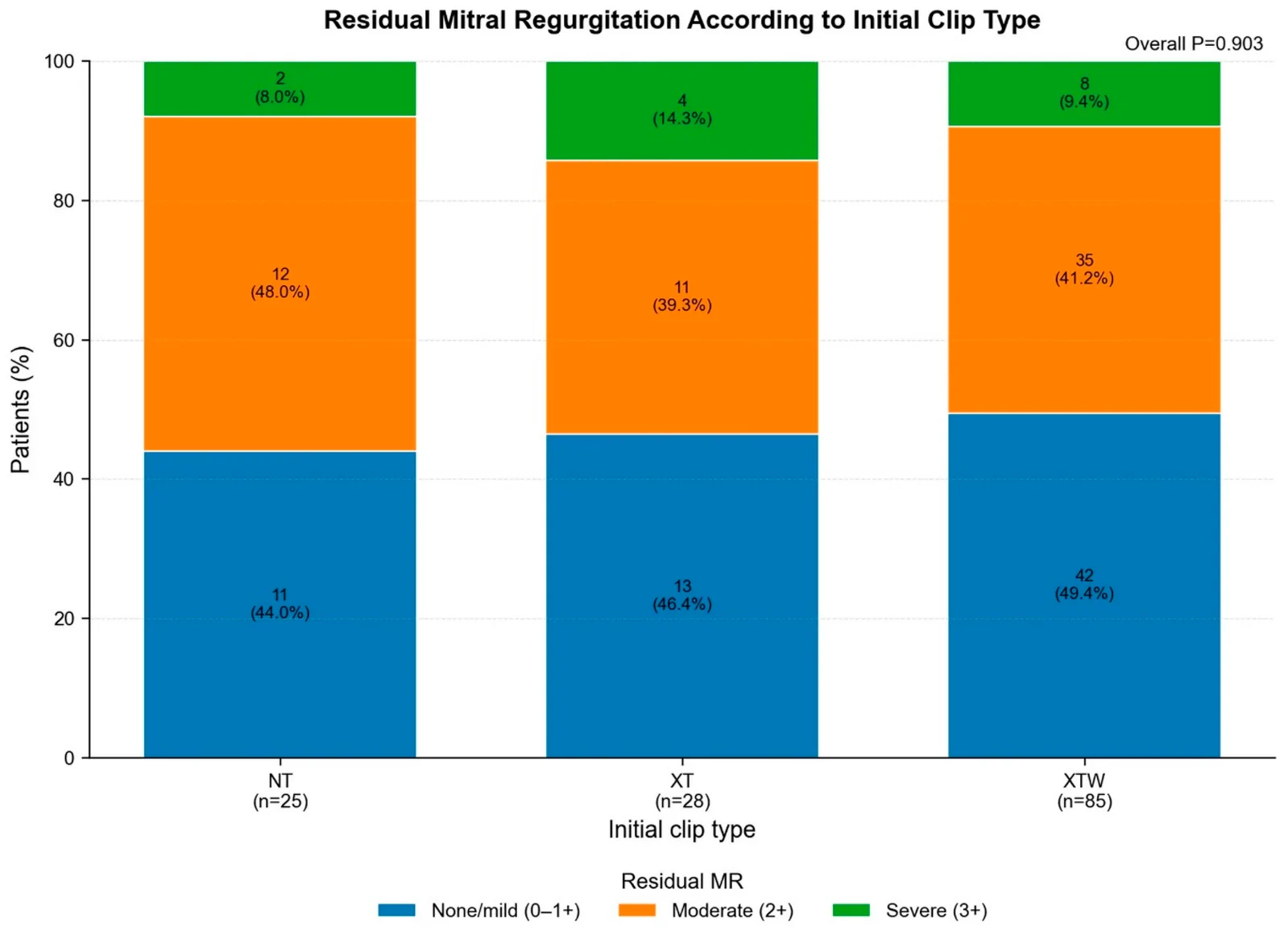
Distribution of pre-discharge residual mitral regurgitation grade according to initial clip type. Values are presented as number (percentage) of patients. MR, mitral regurgitation; NT, narrow standard-length; XT, long narrow-arm; XTW, long wide-arm.

In the multivariable analysis, none of the evaluated variables remained independently associated with residual MR ≥ 2+ in either model (Table 5, Figure 1). No statistically significant association with the outcome was detected for initial XTW clip use in Model 1 (adjusted OR 0.93, 95% CI 0.39–2.18; *p* = 0.861) or for initial NT clip use in Model 2 (adjusted OR 1.66, 95% CI 0.60–4.58; *p* = 0.326); the confidence intervals were wide and do not exclude clinically relevant effects in either direction.

Left atrial diameter showed the strongest trend toward significance in both models (Model 1: adjusted OR 1.22, 95% CI 0.96–1.54, *p* = 0.103; Model 2: adjusted OR 1.22, 95% CI 0.97–1.55, *p* = 0.091), without reaching statistical significance after adjustment. Bootstrap internal validation demonstrated modest model discrimination: apparent AUC was 0.658 for Model 1 and 0.665 for Model 2, decreasing to optimism-corrected values of 0.563 and 0.570, respectively; apparent Brier scores of 0.226 and 0.225 increased to optimism-corrected values of 0.265 and 0.264. All 1000 bootstrap resamples converged successfully, and all VIF values were below 3, indicating no clinically meaningful multicollinearity among predictors. Low VIF values exclude substantial multicollinearity but do not, by themselves, indicate the absence of an effect.

### 3.5. Predictors of Post-Procedural Transmitral Gradient

In multivariable linear regression, secondary (versus primary) MR etiology and higher baseline transmitral mean gradient were independently associated with a higher post-procedural transmitral mean gradient in both models (Table 6, Figure 1). In Model 1, secondary MR was associated with a 0.69 mmHg increase in post-procedural gradient (β = 0.69, 95% CI 0.18–1.20; *p* = 0.008), and each 1 mmHg increase in baseline gradient was associated with a 0.94 mmHg increase post-procedurally (β = 0.94, 95% CI 0.71–1.18; *p* < 0.001); initial XTW clip use was not independently associated with post-procedural gradient (β = −0.12, 95% CI −0.34 to 0.10; *p* = 0.289). Findings were similar in Model 2, with secondary MR (β = 0.65, 95% CI 0.16–1.13; *p* = 0.009) and baseline gradient (β = 0.95, 95% CI 0.70–1.20; *p* < 0.001) remaining independent predictors, while initial NT clip use was not associated with post-procedural gradient (β = 0.00, 95% CI −0.33 to 0.34; *p* = 0.988). LVEF, complex mitral anatomy, EROA, left atrial diameter, moderate-to-severe TR, posterior leaflet length, and number of implanted clips were not independently associated with post-procedural gradient in either model.

### 3.6. Predictors of Requiring ≥2 Clips

In multivariable logistic regression analysis (Supplementary Table S1), none of the evaluated clinical, echocardiographic, or procedural variables were independently associated with the need for implantation of ≥2 clips. Initial XTW clip use was associated with a numerically lower, but not statistically significant, odds of requiring ≥2 clips (adjusted OR 0.51, 95% CI 0.21–1.21; *p* = 0.125), and initial NT clip use was similarly not associated with this outcome (adjusted OR 0.73, 95% CI 0.27–1.99; *p* = 0.538).

### 3.7. Sensitivity Analyses

In sensitivity analyses excluding the total number of implanted clips from the multivariable models, the results remained essentially unchanged. Neither initial XTW nor NT clip selection was significantly associated with residual MR ≥ 2+ (adjusted OR 0.95, 95% CI 0.41–2.22, *p* = 0.911; and adjusted OR 1.45, 95% CI 0.54–3.89, *p* = 0.457, respectively) or post-procedural transmitral mean gradient (β = −0.147, 95% CI −0.366 to 0.072, *p* = 0.188; and β = 0.045, 95% CI −0.305 to 0.395, *p* = 0.800, respectively). Secondary MR remained significantly associated with higher post-procedural mean gradient in both models (XTW model: β = 0.659, 95% CI 0.143–1.175, *p* = 0.012; NT model: β = 0.607, 95% CI 0.119–1.096, *p* = 0.015), as did baseline transmitral mean gradient (XTW model: β = 0.957, 95% CI 0.741–1.172, *p* < 0.001; NT model: β = 0.963, 95% CI 0.727–1.199, *p* < 0.001). No other covariates were significantly associated with either outcome in the sensitivity analyses.

To assess the overall association of initial clip type with procedural outcomes, additional multivariable models were fitted with initial clip type as a three-level categorical variable, using XT as the reference category and excluding the total number of implanted clips. Initial clip type was not significantly associated with residual MR ≥ 2+ (global *p* = 0.713); compared with XT, the adjusted ORs were 1.62 (95% CI 0.50–5.21; *p* = 0.418) for NT and 1.19 (95% CI 0.44–3.26; *p* = 0.731) for XTW. Similarly, initial clip type showed no significant overall association with post-procedural mean transmitral gradient (global *p* = 0.405); compared with XT, the adjusted coefficients were −0.066 mmHg (95% CI −0.610 to 0.477; *p* = 0.811) for NT and −0.178 mmHg (95% CI −0.578 to 0.222; *p* = 0.384) for XTW. Secondary MR and baseline transmitral mean gradient remained significantly associated with post-procedural gradient, whereas the other covariates were not.

To assess the robustness of the initial clip selection models and address potential overfitting, sensitivity analyses were performed using parsimonious models incorporating four clinically relevant anatomical and echocardiographic variables: EROA, LVEF, posterior leaflet length, and complex mitral anatomy. The associations of EROA and LVEF with initial clip selection remained consistent in direction and statistical significance. In the reduced NT model, higher EROA was associated with lower odds of NT selection (OR 0.47, 95% CI 0.28–0.78; *p* = 0.003), whereas higher LVEF was associated with greater odds of NT selection (OR 1.46, 95% CI 1.12–1.90; *p* = 0.005). In the reduced XTW model, higher EROA (OR 2.53, 95% CI 1.61–3.95; *p* < 0.001), lower LVEF (OR 0.70, 95% CI 0.56–0.87; *p* = 0.002), and greater posterior leaflet length (OR 1.43 per 1 mm increase, 95% CI 1.09–1.87; *p* = 0.010) remained significantly associated with XTW selection. Complex anatomy was not significantly associated with clip selection in either reduced model.

### 3.8. Exploratory Follow-Up Outcomes

Follow-up outcomes are reported descriptively only and were not subjected to multivariable or time-to-event analysis. Follow-up was limited to a maximum of 24 months and differed significantly between groups (mean 16.63 ± 10.10 months for NT, 17.58 ± 8.67 months for XT, and 11.74 ± 7.86 months for XTW; *p* = 0.003). Only 15, 17, and 18 patients in the NT, XT, and XTW groups, respectively, reached 24 months of follow-up. Crude all-cause mortality was 48.0%, 39.3%, and 27.1% (*p* = 0.112), and cardiovascular death 28.0%, 28.6%, and 25.9% (*p* = 0.951), in the NT, XT, and XTW groups, respectively. Because of the unequal follow-up and the differences in baseline risk, these crude rates cannot be used to compare clip types and should be regarded as hypothesis-generating only. Mitral valve reintervention was also not significantly different across groups (*p* = 0.639).

## 4. Discussion

In this retrospective, single-center cohort of 138 consecutive patients undergoing mitral TEER, we found that initial clip selection—the choice among narrow, standard-length (NT), long narrow-arm (XT), and long wide-arm (XTW) devices—was systematically guided by anatomic and hemodynamic characteristics of the mitral valve and left ventricle. After adjusting for these characteristics, we found no statistically significant independent association between initial clip type and residual MR, post-procedural transmitral gradient, or the need for additional clips. Given the modest sample size, wide confidence intervals, and limited model discrimination (optimism-corrected AUC 0.563–0.570), this finding should be interpreted as no detectable association in this cohort rather than as evidence that clip geometry has no effect.

### 4.1. Anatomy-Driven Clip Selection

Consistent with the intended design rationale of contemporary TEER platforms, operators in our cohort preferentially selected wider and longer (XTW) clips for patients with larger EROA, more depressed LVEF, longer posterior leaflets, less complex anatomy, and secondary MR, while narrower (NT) clips were reserved for younger patients with smaller regurgitant orifices and preserved LVEF. This pattern mirrors the anatomic reasoning underlying published clip-selection algorithms, which emphasize matching grasping width and arm length to leaflet length, coaptation gap, flail extent, and jet characteristics rather than applying a uniform device strategy across all patients [5]. Similar anatomy-tailored selection has been reported with fourth-generation clip systems offering multiple arm lengths and widths, which have extended treatment to a broader range of leaflet morphologies while maintaining comparable procedural safety and MR reduction across device sizes [4]. The strong, independent association between secondary MR and XTW selection likely reflects the broader coaptation defects and larger effective regurgitant orifices typically seen in functional MR, whereas the association between complex anatomy and lower odds of XTW selection may reflect operator caution in reserving larger devices for straightforward leaflet morphology, or a preference for narrower devices when localized, complex pathology (e.g., a discrete flail segment) is present.

Optimal MR reduction (residual MR ≤ 1+) was achieved in fewer than half of our patients (47.8%), a lower rate than reported in core-laboratory-adjudicated studies of fourth-generation clip systems [4]. Several factors are likely to have contributed. First, our cohort was dominated by secondary MR (81.9%), with advanced left ventricular dysfunction and marked left atrial enlargement, features associated with less complete MR reduction. Second, residual MR was graded by site echocardiographers using a conservative, multiparametric approach, and site-reported MR grades are higher than core-laboratory-adjudicated grades [9]. Third, clips were released when intraprocedural transesophageal echocardiography showed MR ≤ 1+, whereas the endpoint was assessed by predischarge transthoracic echocardiography in awake patients. Reduced afterload under general anesthesia or deep sedation may underestimate MR during the procedure. In addition, acoustic shadowing from the device and eccentric or multiple jets make transthoracic grading more difficult. Importantly, residual MR ≤ 2+ was achieved in 89.9% of patients, and residual MR ≥ 3+ was present in only 10.1%. Nevertheless, because residual MR 2+ has been associated with worse long-term outcomes than residual MR ≤ 1+ [10], the relatively low rate of optimal MR reduction in our cohort indicates room for improvement and should be considered when our findings are interpreted.

Secondary MR was analyzed as a single category in our main models, although atrial and ventricular secondary MR differ in mechanism and anatomy. Atrial functional MR is characterized predominantly by left atrial and annular remodeling with relatively preserved left ventricular geometry, whereas ventricular secondary MR is more closely related to left ventricular remodeling, papillary muscle displacement, leaflet tethering, and reduced coaptation [11]. These differences may influence EROA, effective leaflet length, and coaptation geometry, and therefore both clip selection and the procedural response to TEER. However, the small number of patients with atrial secondary MR (*n* = 31) limits the precision of these estimates, and the question should be addressed in larger cohorts.

In addition, MR is a dynamic phenomenon that varies throughout systole, whereas vena contracta width and PISA are usually derived from a single systolic frame. These measurements may therefore not fully reflect the temporal and three-dimensional complexity of the regurgitant orifice, and may lead to inaccurate estimation of MR severity in selected patients [12,13]. Therefore, integrating thoracic anatomy, multiparametric and time-resolved Doppler assessment, volumetric methods and, when available, three-dimensional measurements such as 3D vena contracta area may help reduce MR overestimation and improve patient selection and procedural planning for mitral TEER [13].

Because baseline EROA was a major determinant of clip selection, factors affecting MR quantification may also influence procedural planning. A concave-shaped chest wall may alter cardiac position and mitral geometry through extrinsic compression, and MR severity may be overestimated in some of these patients [14]. However, this evidence derives mainly from primary MR, whereas most of our patients had secondary MR.

### 4.2. No Detectable Independent Association Between Clip Type and Echocardiographic Outcome

Despite these clear selection patterns, no statistically significant association was detected between initial clip type and residual MR ≥ 2+, post-procedural transmitral gradient, or the need for a second clip after adjustment for baseline anatomy and hemodynamics. On univariable analysis, residual MR ≥ 2+ was associated with markers of atrial and right-heart burden—left atrial diameter, left atrial area, and moderate-to-severe TR—although none of these remained significant after multivariable adjustment. Post-procedural gradient elevation was associated with secondary MR etiology and a higher baseline transmitral gradient, both of which plausibly reflect pre-existing valvular and annular characteristics (e.g., annular dilatation, reduced mitral valve area) rather than the specific grasping geometry of the implanted device.

Taken together, these findings are consistent with the possibility that outcomes do not differ substantially by clip type when the initial device is deliberately chosen to match leaflet and orifice characteristics. This concept broadly aligns with real-world registry data on initial clip selection [15] and with editorial and review data emphasizing that no single leaflet pathology or anatomic pattern (anterior, posterior, or bileaflet) should preclude a tailored M-TEER strategy [9]. However, our study was not designed or powered to demonstrate equivalence between clip strategies, and this interpretation should be regarded as a hypothesis derived from observational data.

This observation is relevant because residual MR after TEER is a well-established determinant of downstream outcomes, including heart failure hospitalization and survival, in both degenerative and functional MR [3,16]. In our cohort, residual MR was associated with markers of atrial and right-heart remodeling on univariable analysis, whereas it did not differ significantly by the specific clip implanted. Because clip type was chosen according to anatomy, this observation describes outcomes within an anatomy-guided selection practice and should not be interpreted as evidence that clip choice does not affect procedural efficacy; modest differences between clip types cannot be excluded. Similarly, no significant association was found between the number of clips and residual MR, and the numerically lower proportion of XTW patients requiring a second clip did not reach statistical significance. This finding may be compatible with, but does not test, the hypothesis that appropriately sized initial clips reduce the need for supplemental grasping; our sample size likely limited the power to detect a modest effect.

### 4.3. Comparison with the OCEAN-Mitral Registry

Our findings are broadly concordant with, and help contextualize, the largest available real-world dataset addressing this question. In the OCEAN-Mitral registry of 2257 patients receiving the MitraClip G4, initial clip type was distributed as NT 18.9%, NTW 41.7%, XT 5.1%, and XTW 34.3% [15]; in our cohort, the corresponding first-clip distribution was NT 18.1%, XT 20.3%, and XTW 61.6%. Because NTW was not used as the initial device in our cohort, category-by-category comparison with OCEAN-Mitral should be interpreted with caution. When devices are grouped by arm length, long clips (XT or XTW) were used initially in 81.9% of our patients compared with 39.4% in OCEAN-Mitral, whereas wide clips (NTW or XTW) were used in 61.6% and 76.0%, respectively. Our center therefore differed mainly in its preference for long arms, which may reflect the higher proportion of secondary MR and more depressed LVEF in our population, as well as operator preference.

As in our cohort, Kubo et al. found that the proportion of patients achieving MR severity ≤ 1+ did not differ significantly among the four clip types at either discharge or 1-year follow-up, and that death or heart failure hospitalization did not differ significantly between long versus short or wide versus narrow clip strategies [15].

This consistency with a registry approximately 16 times larger is noteworthy; however, neither study was designed to establish equivalence, and an effect of clip geometry in specific subgroups, such as patients with primary MR, cannot be excluded. Two nuances from the OCEAN-Mitral experience merit comment. First, after propensity matching for anatomical features, Kubo et al. found that an initial long-clip strategy was associated with a greater degree of MR reduction and a higher proportion of MR ≤ 1+ at 1 year specifically in patients with primary MR, whereas no such benefit was observed in secondary MR [15]; our study, which included fewer primary-MR patients and did not perform an etiology-stratified propensity-matched comparison of clip geometry, may therefore have been underpowered to detect an analogous primary MR-specific effect of arm length. This represents a natural next analysis for our dataset. Second, the OCEAN-Mitral registry reported no meaningful excess of leaflet tear or single-leaflet device attachment with long or wide clips, and the few leaflet tears that did occur with long clips were concentrated in secondary rather than primary MR [15]. In our cohort, single-leaflet device attachment (7.1% vs. 3.6% vs. 0%) and major bleeding (16.5% vs. 7.1% vs. 4.0%) were numerically more frequent in the XTW group; however, these differences were not statistically significant, and the small number of events precludes any conclusion regarding the comparative safety of clip types.

### 4.4. Model Performance and Interpretation

The modest discrimination of our multivariable models for residual MR ≥ 2+ (optimism-corrected AUC 0.563–0.570) indicates that the anatomic and procedural variables captured in this dataset explain only a limited proportion of the variability in procedural echocardiographic outcome. This is consistent with the recognized complexity of the mitral apparatus and with contemporary reviews of M-TEER, in which residual MR is influenced by factors difficult to fully capture in a retrospective dataset—including three-dimensional leaflet and annular geometry, real-time grasping quality, operator experience, and intraprocedural hemodynamic conditions [10,17]. Together with the modest discrimination and wide confidence intervals, the null findings for clip type should be regarded as inconclusive rather than as evidence of no effect.

### 4.5. Clinical Implications

Our data describe how initial clips were selected in routine practice: wider and longer clips were chosen for larger regurgitant orifices, lower LVEF, longer posterior leaflets and secondary MR, and narrower clips for smaller orifices and preserved LVEF. Within this anatomy-guided practice, no statistically significant differences in residual MR, post-procedural gradient, or the need for additional clips were detected between initial clip types. Because of the observational design and confounding by indication, these data cannot establish the superiority or equivalence of any clip-selection strategy, and they do not show that clip types are interchangeable. Prospective studies, ideally with standardized selection protocols and core-laboratory adjudication, are needed to define whether and in which anatomies clip geometry influences outcomes [18,19].

### 4.6. Limitations

Several limitations should be considered. First, this was a retrospective, single-center, observational study without randomization of clip type, and unmeasured or imperfectly captured confounders—including 3D leaflet/annular geometry, real-time grasping quality, and operator experience—may have influenced both clip selection and outcome. Second, the relatively modest and unevenly distributed sample size across clip-type subgroups (NT, *n* = 25; XT, *n* = 28; XTW, *n* = 85) limited statistical power, particularly for the NT and XT groups, and produced wide confidence intervals around several point estimates; some borderline associations (e.g., left atrial diameter, posterior leaflet length, or the reduced odds of requiring ≥2 clips with XTW) may reflect true but underpowered effects rather than true null findings. Furthermore, the relatively low events-per-parameter ratio (71 events for nine predictor parameters in the residual MR ≥ 2+ models [7.89 per parameter, below the commonly recommended threshold of 10], and 25 events [2.78 per parameter] in the NT-selection model) raises concerns regarding potential overfitting and model instability. The reduction in AUC after bootstrap correction (from 0.658 to 0.563 in Model 1 and from 0.665 to 0.570 in Model 2) further indicates limited generalizability, warranting cautious interpretation of the multivariable findings. Notably, our sample size is roughly 6% of the OCEAN-Mitral registry [15], and we could not replicate its propensity-matched, etiology-stratified design; a primary-MR-specific benefit of longer clips, such as that reported in OCEAN-Mitral, therefore cannot be excluded by our data. Third, an independent core laboratory did not adjudicate echocardiographic measurements, interobserver variability for residual MR grading and transmitral gradient was not assessed, and follow-up echocardiography was performed at the discretion of treating physicians rather than at fixed intervals, introducing potential measurement and ascertainment variability. Grading residual MR after TEER is particularly prone to interobserver variability, and site-based grading may have overestimated residual MR severity—a limitation shared with most real-world TEER registries, including OCEAN-Mitral, in which site-reported MR severity has been noted to run higher than core-laboratory-adjudicated rates [15]. Because the same protocol was applied to all patients, this misclassification is likely to be similar across clip groups. Fourth, our multivariable models for residual MR and post-procedural gradient showed only modest discrimination, indicating that other unmeasured determinants of procedural outcome remain to be identified. Fifth, our models for residual MR and post-procedural gradient adjusted for the number of implanted clips; because additional clips were placed in response to residual MR judged insufficient during the same procedure, this variable is more accurately characterized as a potential mediator or procedure-concurrent covariate than a true baseline confounder, and its inclusion may have attenuated the apparent association between initial clip type and these outcomes. Sensitivity analyses excluding this covariate yielded essentially unchanged results.

Baseline vena contracta width and PISA-derived EROA were obtained from single-frame 2D measurements and may not fully capture the dynamic and three-dimensional nature of MR [12]. In addition, 3D vena contracta area, which may better quantify residual MR after TEER, was not systematically available in this retrospective cohort [20].

Chest wall conformation was not assessed in this cohort; therefore, its possible influence on MR grading and clip selection cannot be excluded [14].

Because initial clip selection was based on operator judgment and valve anatomy, substantial confounding by indication is likely and cannot be fully removed by multivariable adjustment. Multinomial and propensity-based analyses were not feasible because of the small NT and XT groups. Our findings should therefore be interpreted as associations observed within an anatomy-tailored selection strategy rather than as evidence of equivalence between clip geometries.

The relatively small sample size, particularly in the NT and XT groups, and the number of covariates included in the multivariable models introduce a potential risk of overfitting and unstable estimates. This concern is particularly relevant to the initial NT selection model, which included 25 events and nine predictor parameters. Although all models converged and no substantial multicollinearity was identified, these findings should be interpreted as exploratory and require validation in larger cohorts.

The limited number of patients with primary MR precluded reliable interaction or stratified analyses to determine whether associations between initial clip type and procedural outcomes differed according to MR etiology. Furthermore, ventricular and atrial secondary MR were analyzed together despite their distinct pathophysiological and anatomical characteristics. Potential differences in the effects of clip selection across these etiological subgroups therefore remain to be investigated in larger cohorts.

The relatively small numbers of patients receiving NT and XT clips limited the statistical precision of comparisons among the three clip categories. Although additional multivariable models treating initial clip type as a three-level categorical variable were performed for procedural outcomes, the determinants of initial clip selection were assessed using separate one-versus-rest logistic regression models rather than multinomial logistic regression because of the limited sample sizes in the NT and XT groups. Consequently, these exploratory analyses do not provide a unified assessment of all three clip categories, and their findings should be interpreted cautiously.

Finally, mortality and rehospitalization outcomes were reported as crude, unadjusted rates and should be regarded as descriptive and hypothesis-generating observations only. Follow-up duration differed significantly among clip groups, with a shorter median follow-up in the XTW group (10.32 months) than in the NT and XT groups (24.00 months each). Furthermore, only 15, 17, and 18 patients in the NT, XT, and XTW groups, respectively, had reached 24 months of follow-up. Given the unequal observation periods and limited number of patients with longer-term follow-up, particularly in the XTW group, formal time-to-event analyses were not undertaken. Mortality after TEER is influenced by numerous factors beyond initial clip geometry, including MR etiology, residual MR, comorbidity burden, and frailty [3,21,22,23,24]. Therefore, the observed crude mortality and rehospitalization rates should not be interpreted as evidence of differences or equivalence among clip types.

Importantly, the non-significant associations between initial clip type and outcomes should not be interpreted as evidence of equivalence; the confidence intervals remain compatible with clinically relevant effects in either direction.

Larger prospective studies with more complete and balanced follow-up are needed to evaluate long-term clinical outcomes using appropriately adjusted time-to-event methods.

## 5. Conclusions

In this real-world, single-center cohort of patients undergoing mitral TEER, initial clip selection was guided by mitral valve anatomy, LVEF, and MR etiology. Within this anatomy-guided selection strategy, no statistically significant independent association was detected between initial clip type and residual MR ≥ 2+, post-procedural transmitral gradient, or the need for additional clips. Because operators selected different devices for different anatomies, these findings are subject to confounding by indication; they do not establish that clip geometry has no effect, nor that one selection strategy is superior or equivalent to another. Larger prospective multicenter studies with core-laboratory echocardiographic adjudication are required to determine whether clinically relevant differences between clip geometries exist.

## Figures and Tables

**Table 1:** Baseline, echocardiographic, procedural, and clinical outcome characteristics according to the first implanted clip type.

Variable	NT (*n* = 25)	XT (*n* = 28)	XTW (*n* = 85)	*p* Value
Baseline Clinical Characteristics				
Age, years	74.00 (66.00–80.00)	74.50 (66.00–82.50)	75.00 (70.00–79.00)	0.827
Male sex	13 (52.0%)	21 (75.0%)	57 (67.1%)	0.199
Hypertension	17 (68.0%)	23 (82.1%)	69 (81.2%)	0.328
Diabetes mellitus	6 (24.0%)	4 (14.3%)	21 (24.7%)	0.508
Coronary artery disease	20 (80.0%)	21 (75.0%)	68 (80.0%)	0.845
Atrial fibrillation	15 (60.0%)	13 (46.4%)	41 (48.2%)	0.536
Peripheral artery disease	3 (12.0%)	0 (0.0%)	4 (4.7%)	0.133
Previous CABG	5 (20.0%)	9 (32.1%)	12 (14.1%)	0.105
Cardiac resynchronization therapy	5 (20.0%)	4 (14.3%)	19 (22.4%)	0.654
Chronic kidney disease	11 (44.0%)	12 (42.9%)	48 (56.5%)	0.326
Previous heart failure hospitalization	9 (36.0%)	14 (50.0%)	42 (49.4%)	0.469
**Baseline medical therapy**				
ACE inhibitor or ARB use	11 (44.0%)	9 (32.1%)	37 (43.5%)	0.544
ARNI use	5 (20.0%)	15 (53.6%)	21 (24.7%)	0.008
Diuretic use	25 (100.0%)	27 (96.4%)	74 (87.1%)	0.080
Beta-blocker use	23 (92.0%)	26 (92.9%)	74 (87.1%)	0.641
Mineralocorticoid receptor antagonist use	15 (60.0%)	20 (71.4%)	39 (45.9%)	0.049
SGLT2 inhibitor use	5 (20.0%)	14 (50.0%)	35 (41.2%)	0.068
**Laboratory parameters**				
Baseline creatinine (mg/dL)	1.10 (0.90–1.50)	0.90 (0.83–1.40)	1.20 (0.90–1.50)	0.576
Postprocedural creatinine (mg/dL)	1.10 (0.90–1.80)	1.00 (0.90–1.70)	1.30 (0.95–1.70)	0.439
Hemoglobin (g/dL)	11.46 ± 2.50	12.26 ± 1.79	11.50 ± 1.64	0.146
Estimated glomerular filtration rate (mL/min/1.73 m^2^)	57.00 (38.00–85.00)	63.50 (39.25–92.00)	55.75 (37.80–65.00)	0.279
NT-proBNP (pg/mL)	2775.00 (817.00–4150.50)	13,727.00 (11,900.00–13,727.00)	8896.50 (3444.50–21,513.50)	0.014
**Mitral regurgitation etiology and anatomical characteristics**				
Mitral regurgitation etiology				0.317
Primary MR	6 (24.0%)	5 (17.9%)	14 (16.5%)	
Ventricular secondary MR	11 (44.0%)	15 (53.6%)	56 (65.9%)	
Atrial secondary MR	8 (32.0%)	8 (28.6%)	15 (17.6%)	
Complex mitral anatomy	6 (24.0%)	9 (32.1%)	11 (12.9%)	0.061
Mitral annular calcification	2 (8.0%)	1 (3.6%)	3 (3.5%)	0.728
Effective regurgitant orifice area (mm^2^)	45.00 (43.00–50.00)	43.00 (41.75–50.00)	50.00 (46.00–55.00)	0.003
Posterior leaflet length (mm)	1.10 (1.10–1.20)	1.10 (1.10–1.20)	1.20 (1.10–1.30)	0.211
Baseline transmitral mean gradient (mmHg)	3.00 (3.00–4.00)	3.10 (3.00–4.00)	3.00 (2.08–4.00)	0.132
**Baseline echocardiographic characteristics**				
Left ventricular end-diastolic diameter (mm)	56.00 (54.00–63.00)	60.00 (54.75–67.75)	57.00 (53.00–63.00)	0.203
Left ventricular end-systolic diameter (mm)	42.00 (37.00–53.00)	48.50 (40.75–55.50)	45.00 (38.00–53.00)	0.376
Left ventricular ejection fraction (%)	35.00 (30.00–50.00)	30.00 (25.00–45.00)	40.00 (25.00–45.00)	0.512
Left atrial diameter (mm)	48.00 (43.00–52.00)	49.00 (46.75–55.25)	48.00 (44.00–55.00)	0.163
Left atrial area (cm^2^)	25.00 (22.00–28.00)	26.50 (24.00–30.00)	27.00 (24.00–30.00)	0.396
Baseline pulmonary artery systolic pressure (mmHg)	50.60 ± 14.02	51.25 ± 15.91	49.94 ± 13.44	0.908
Moderate-to-severe tricuspid regurgitation	20 (80.0%)	26 (92.9%)	71 (83.5%)	0.350

Abbreviations: ACE, angiotensin-converting enzyme; ARB, angiotensin receptor blocker; ARNI, angiotensin receptor–neprilysin inhibitor; CABG, coronary artery bypass grafting; MR, mitral regurgitation; NT-proBNP, N-terminal pro-B-type natriuretic peptide; SGLT2, sodium–glucose cotransporter 2. *p*-values are unadjusted for multiple comparisons and are presented for descriptive purposes only. Bold is used to mark the subgroup headings within the table.

**Table 2:** Procedural and Postprocedural Characteristics, In-Hospital Complications, and Follow-Up Outcomes According to the First Implanted Clip Type.

Procedural and Postprocedural Characteristics	NT (*n* = 25)	XT (*n* = 28)	XTW (*n* = 85)	
Total number of clips	1.72 ± 0.74	1.89 ± 0.50	1.64 ± 0.67	0.195
Postprocedural MR grade 0–1	11 (44.0%)	13 (46.4%)	42 (49.4%)	0.881
Postprocedural MR grade 2	12 (48.0%)	11 (39.3%)	35 (41.2%)	0.788
Postprocedural MR grade ≥ 3	2 (8.0%)	4 (14.3%)	8 (9.4%)	0.722
Postprocedural transmitral mean gradient (mmHg)	3.50 (3.00–4.10)	3.10 (3.00–4.03)	3.00 (2.50–4.00)	0.211
Postprocedural pulmonary artery systolic pressure (mmHg)	50.00 (40.00–55.00)	45.00 (38.75–50.00)	40.00 (35.00–50.00)	0.131
Postprocedural tricuspid regurgitation grade				0.380
Grade 1	6 (24.0%)	4 (14.3%)	30 (35.3%)	
Grade 2	10 (40.0%)	15 (53.6%)	32 (37.6%)	
Grade 3	9 (36.0%)	8 (28.6%)	21 (24.7%)	
Grade 4	0 (0.0%)	1 (3.6%)	2 (2.4%)	
Single leaflet device attachment or detachment	0 (0.0%)	1 (3.6%)	6 (7.1%)	0.427
**In-hospital renal and procedural complications**				
Acute kidney injury	8 (32.0%)	9 (32.1%)	35 (41.2%)	0.562
Need for dialysis	4 (16.0%)	3 (10.7%)	15 (17.6%)	0.722
Cerebrovascular accident	2 (8.0%)	0 (0.0%)	10 (11.8%)	0.165
Major bleeding	1 (4.0%)	2 (7.1%)	14 (16.5%)	0.187
Peripheral vascular complication	0 (0.0%)	0 (0.0%)	4 (4.7%)	0.469
Need for emergent surgery	0 (0.0%)	0 (0.0%)	0 (0.0%)	1.000
Procedural death	0 (0.0%)	0 (0.0%)	0 (0.0%)	1.000
**Clinical outcomes during follow-up**				
All-cause mortality	12 (48.0%)	11 (39.3%)	23 (27.1%)	0.112
Cardiovascular death	7 (28.0%)	8 (28.6%)	22 (25.9%)	0.951
Heart failure rehospitalization	7 (28.0%)	4 (14.3%)	32 (37.6%)	0.064
Mitral valve reintervention	1 (4.0%)	0 (0.0%)	3 (3.5%)	0.639

Abbreviations: MR: mitral regurgitation.

**Table 3:** Multivariable logistic regression analysis of factors associated with initial clip selection.

Variable	XTW vs. Other Clip Types, Adjusted OR (95% CI)	*p* Value	NT vs. Other Clip Types, Adjusted OR (95% CI)	*p* Value
Age, per 5-year increase	1.243 (0.972–1.590)	0.083	0.768 (0.590–0.999)	**0.049**
Male sex	1.259 (0.501–3.162)	0.624	0.426 (0.151–1.202)	0.107
EROA, per 5 mm^2^ increase	3.869 (2.215–6.759)	**<0.001**	0.341 (0.182–0.640)	**<0.001**
LVEF, per 5-percentage-point increase	0.727 (0.565–0.934)	**0.013**	1.440 (1.063–1.949)	**0.019**
Posterior leaflet length, per 1 mm increase	1.526 (1.116–2.086)	**0.008**	0.706 (0.489–1.020)	0.064
Complex mitral anatomy	0.255 (0.079–0.825)	**0.023**	1.655 (0.461–5.941)	0.440
Secondary MR vs. primary MR	12.684 (2.559–62.868)	**0.002**	0.163 (0.025–1.077)	0.060
Baseline transmitral mean gradient, per 1 mmHg increase	0.726 (0.504–1.045)	0.085	1.274 (0.840–1.934)	0.254
Mitral annular calcification	1.056 (0.146–7.655)	0.957	1.768 (0.250–12.490)	0.568

Abbreviations: EROA, effective regurgitant orifice area; LVEF, left ventricular ejection fraction; MR, mitral regurgitation; OR, odds ratio. Bold values indicate statistical significance (*p* < 0.05).

**Table 4:** Univariable logistic regression analysis of factors associated with residual mitral regurgitation ≥ 2+ after mitral transcatheter edge-to-edge repair.

Variable	Unadjusted OR (95% CI)	*p* Value
Age, per 5-year increase	1.06 (0.89–1.26)	0.527
Male sex	0.83 (0.41–1.67)	0.595
Log-transformed NT-proBNP	1.08 (0.53–2.21)	0.834
Secondary versus primary mitral regurgitation	1.82 (0.76–4.40)	0.182
Complex mitral anatomy	1.96 (0.81–4.76)	0.139
EROA, per 5 mm^2^ increase	0.88 (0.69–1.12)	0.296
LVEDD, per 5 mm increase	1.03 (0.85–1.25)	0.757
LVESD, per 5 mm increase	1.13 (0.95–1.33)	0.161
LVEF, per 5-percentage-point increase	0.96 (0.85–1.08)	0.495
**Left atrial diameter**, per 5 mm increase	**1.31 (1.05–1.64)**	**0.016**
**Left atrial area**, per 5 cm^2^ increase	**1.46 (1.00–2.11)**	**0.047**
**Moderate-to-severe tricuspid regurgitation**	**3.20 (1.16–8.84)**	**0.025**
Baseline transmitral mean gradient, per 1 mmHg increase	1.10 (0.84–1.45)	0.490
Posterior leaflet length, per 1 mm increase	1.03 (0.85–1.25)	0.759
Mitral annular calcification	0.91 (0.18–4.69)	0.913
Number of implanted clips, per 1-clip increase	1.35 (0.81–2.28)	0.254
Initial XTW clip versus other clip types	0.85 (0.43–1.69)	0.637
Initial NT clip versus other clip types	1.21 (0.51–2.89)	0.672

Abbreviations: CI, confidence interval; EROA, effective regurgitant orifice area; LVEDD, left ventricular end-diastolic diameter; LVEF, left ventricular ejection fraction; LVESD, left ventricular end-systolic diameter; NT, narrow standard-length; NT-proBNP, N-terminal pro-B-type natriuretic peptide; OR, odds ratio; XTW, long wide-arm. Bold values indicate statistical significance (*p* < 0.05).

**Table 5:** Multivariable logistic regression analysis of factors associated with residual mitral regurgitation ≥ 2+ after mitral transcatheter edge-to-edge repair.

Variable	Model 1, Adjusted OR (95% CI)	*p* Value	Model 2, Adjusted OR (95% CI)	*p* Value
LVEF, per 5-percentage-point increase	1.03 (0.85–1.25)	0.761	1.02 (0.84–1.23)	0.874
Secondary versus primary mitral regurgitation	1.42 (0.37–5.37)	0.607	1.50 (0.41–5.55)	0.540
Complex mitral anatomy	1.92 (0.69–5.37)	0.213	1.96 (0.70–5.46)	0.199
EROA, per 5 mm^2^ increase	0.87 (0.57–1.34)	0.531	0.91 (0.61–1.35)	0.629
Left atrial diameter, per 5 mm increase	1.22 (0.96–1.54)	0.103	1.22 (0.97–1.55)	0.091
Moderate-to-severe tricuspid regurgitation	2.27 (0.76–6.73)	0.141	2.39 (0.80–7.18)	0.120
Baseline transmitral mean gradient, per 1 mmHg increase	1.04 (0.76–1.42)	0.798	1.02 (0.74–1.39)	0.912
Posterior leaflet length, per 1 mm increase	1.02 (0.82–1.26)	0.876	1.03 (0.83–1.27)	0.808
Number of implanted clips, per 1-clip increase	1.10 (0.61–1.99)	0.747	1.11 (0.62–1.99)	0.732
Initial XTW clip versus other clip types	0.93 (0.39–2.18)	0.861	—	—
Initial NT clip versus other clip types	—	—	1.66 (0.60–4.58)	0.326

Abbreviations: CI, confidence interval; EROA, effective regurgitant orifice area; LVEF, left ventricular ejection fraction; NT, narrow standard-length; OR, odds ratio; XTW, long wide-arm.

**Table 6:** Multivariable linear regression analysis for predictors of post-procedural transmitral mean gradient.

Variable	Model 1 Initial XTW vs. Other Clip Types Adjusted β (95% CI)	*p* Value	Model 2 Initial NT vs. Other Clip Types Adjusted β (95% CI)	*p* Value
LVEF, per 5-percentage-point increase	0.05 (−0.06 to 0.16)	0.366	0.06 (−0.07 to 0.18)	0.369
Secondary vs. primary mitral regurgitation	**0.69 (0.18 to 1.20)**	**0.008**	**0.65 (0.16 to 1.13)**	**0.009**
Complex mitral anatomy	−0.18 (−0.60 to 0.25)	0.416	−0.15 (−0.60 to 0.30)	0.509
EROA, per 5 mm^2^ increase	0.11 (−0.02 to 0.24)	0.095	0.09 (−0.08 to 0.25)	0.318
Left atrial diameter, per 5 mm increase	0.04 (−0.09 to 0.17)	0.522	0.04 (−0.09 to 0.17)	0.511
Moderate-to-severe tricuspid regurgitation	0.03 (−0.32 to 0.37)	0.875	0.02 (−0.34 to 0.39)	0.892
Baseline transmitral mean gradient, per 1 mmHg increase	**0.94 (0.71 to 1.18)**	**<0.001**	**0.95 (0.70 to 1.20)**	**<0.001**
Posterior leaflet length, per 1 mm increase	0.04 (−0.02 to 0.10)	0.175	0.04 (−0.02 to 0.09)	0.247
Number of implanted clips, per 1-clip increase	0.10 (−0.17 to 0.38)	0.465	0.11 (−0.16 to 0.39)	0.426
Initial XTW clip vs. other clip types	−0.12 (−0.34 to 0.10)	0.289	—	—
Initial NT clip vs. other clip types	—	—	0.00 (−0.33 to 0.34)	0.988

Abbreviations: β, regression coefficient; CI, confidence interval; EROA, effective regurgitant orifice area; LVEF, left ventricular ejection fraction; NT, narrow standard-length; XTW, long wide-arm. Bold values indicate statistical significance (*p* < 0.05).

## Data Availability

The data presented in this study are available on reasonable request from the corresponding author. The data are not publicly available due to patient privacy and ethical restrictions.

## Supplementary Materials

The following supporting information can be downloaded at: https://www.mdpi.com/article/10.3390/jcm15208176/s1, Table S1: Multivariable logistic regression analysis of factors associated with the need for ≥2 clips.
