# Supplementary material for: Initial Clip Selection and Outcomes After Mitral Transcatheter Edge-to-Edge Repair: A Single-Center Experience
Source: J Clin Med. 2026 Oct 21;15(20):8176. doi: 10.3390/jcm15208176 (PMC13705671; doi:10.3390/jcm15208176)
Supplement: Supplementary file 1 [file jcm-15-08176-s001.zip › jcm-4591552-supplementary.pdf]

Supplementary Table S1. Multivariable logistic regression analysis for predictors of requirement for ≥2 clips during mitral transcatheter edge-to-edge repair

Variable	Model 1 Initial XTW vs other clip types Adjusted OR (95% CI)	p value	Model 2 Initial NT vs other clip types Adjusted OR (95% CI)	p value
LVEF, per 5-percentage-point increase	0.89 (0.73-1.09)	0.266	0.93 (0.76-1.14)	0.489
Secondary vs primary mitral regurgitation	0.64 (0.16-2.50)	0.517	0.46 (0.12-1.73)	0.248
Complex mitral anatomy	1.85 (0.64-5.35)	0.257	2.07 (0.73-5.85)	0.168
EROA, per 5-unit increase	1.15 (0.75-1.77)	0.517	0.96 (0.64-1.43)	0.839
Left atrial diameter, per 5-unit increase	1.12 (0.89-1.42)	0.333	1.13 (0.89-1.44)	0.316
Moderate-to-severe tricuspid regurgitation	2.20 (0.78-6.20)	0.136	2.08 (0.74-5.83)	0.165
Baseline transmitral mean gradient, per 1-mmHg increase	1.19 (0.86-1.65)	0.294	1.27 (0.92-1.76)	0.148
Posterior leaflet length, per 1-mm increase	1.09 (0.88-1.35)	0.442	1.05 (0.85-1.30)	0.667
Initial XTW clip vs other clip types	0.51 (0.21-1.21)	0.125	—	—
Initial NT clip vs other clip types	—	—	0.73 (0.27-1.99)	0.538

Abbreviations: CI, confidence interval; EROA, effective regurgitant orifice area; LVEF, left ventricular ejection fraction; NT, narrow MitraClip; OR, odds ratio; XTW, extended-wide MitraClip.
